# A computational model for potential microbe–disease association detection based on improved graph convolutional networks and multi-channel autoencoders

**DOI:** 10.3389/fmicb.2024.1435408

**Published:** 2024-07-29

**Authors:** Chuyi Zhang, Zhen Zhang, Feng Zhang, Bin Zeng, Xin Liu, Lei Wang

**Affiliations:** Big Data Innovation and Entrepreneurship Education Center of Hunan Province, Changsha University, Changsha, China

**Keywords:** graph attention networks, sparse autoencoders, microbe–disease associations, computational model, prediction model

## Abstract

**Introduction:**

Accumulating evidence shows that human health and disease are closely related to the microbes in the human body.

**Methods:**

In this manuscript, a new computational model based on graph attention networks and sparse autoencoders, called GCANCAE, was proposed for inferring possible microbe–disease associations. In GCANCAE, we first constructed a heterogeneous network by combining known microbe–disease relationships, disease similarity, and microbial similarity. Then, we adopted the improved GCN and the CSAE to extract neighbor relations in the adjacency matrix and novel feature representations in heterogeneous networks. After that, in order to estimate the likelihood of a potential microbe associated with a disease, we integrated these two types of representations to create unique eigenmatrices for diseases and microbes, respectively, and obtained predicted scores for potential microbe–disease associations by calculating the inner product of these two types of eigenmatrices.

**Results and discussion:**

Based on the baseline databases such as the HMDAD and the Disbiome, intensive experiments were conducted to evaluate the prediction ability of GCANCAE, and the experimental results demonstrated that GCANCAE achieved better performance than state-of-the-art competitive methods under the frameworks of both 2-fold and 5-fold CV. Furthermore, case studies of three categories of common diseases, such as asthma, irritable bowel syndrome (IBS), and type 2 diabetes (T2D), confirmed the efficiency of GCANCAE.

## 1 Introduction

Microorganisms are very important to human health (Gill et al., 2006; Integrative HMP (iHMP) Research Network Consortium, 2014; Proctor et al., 2019). Human body is inhabited by a vast number of microorganisms which form a complex ecological community and influence the human physiology in the aspect of both health and diseases (Dekaboruah et al., 2020). The interplay between the commensal microbiota and the mammalian immune system development and function includes multifold interactions in homeostasis and disease (Zheng, 2020). Moreover, microbiome may contribute to immune dysfunction of human body and the emergence of human diseases (Shi et al., 2017), changes in the composition of microbiota may be linked to the pathogenesis of different neurological disorders (Kim et al., 2018), and almost all digestive tract diseases are related to the gut microbiota (Kim et al., 2019). In recent years, research studies show that microbiota is closely related to the development and progression of human gastrointestinal diseases (Ohkusa et al., 2002), cancers (Luu et al., 2017), neurodegenerative diseases (Sampson et al., 2016), and cardiovascular diseases (Toya et al., 2020). Certainly, microbes can help to improve human health. For instance, numerous clinical studies have reported that prebiotics, or probiotics, can reduce symptoms of autism, depression, and other neurological disorders of human body (Guarner and Malagelada, 2003). Moreover, a simple approach for creating new treatments for complicated illnesses of the central nervous system may be the modification of microbiota (Cryan and Dinan, 2012; El-Sayed et al., 2021). Furthermore, it has been demonstrated that the microbiome and its particular metabolites may contribute to the pathophysiology and/or development of a number of human diseases (Illiano et al., 2020).

In the past few years, due to the rapid development of high-throughput sequencing technologies and advanced histological methods, numerous databases of known microbe–disease association have been created by worldwide researchers for further exploring potential connections between microbes and diseases. For instance, Ma et al. established a microbe–disease association database called HMDAD by gathering 483 known associations between 39 diseases and 292 microorganisms from 61 academic papers in 2016 (Ma et al., 2017). In 2018, a new microbe–disease association database named Disbiome was built by Janssens et al. (2018) through compiling 10,922 known associations between 372 diseases and 1,622 microorganisms from experimental records of 1,191 published literature studies. Based on these two databases, in 2020, Yao et al. created another more complicated microbe–disease association database known as MicroPhenoDB, which contains 696,934 known associations between 27,277 branch-specific core genes and 685 microorganisms and 5,677 known associations between 1,781 microbes and 542 human disease phenotypes extracted from 22 newly collected human sections (Yao et al., 2020). In 2021, Wu et al. built a novel microbe–disease association database called MDIDB by selecting 44,900 known associations between 1,065 microorganisms and 1,198 diseases from 8,458 publications (Wu et al., 2021). In addition, G. skoufos et al. constructed a Peryton-based microbe–disease association database in 2021 by collecting 7,977 known associations between 43 diseases and 1,396 microorganisms from 314 academic articles (Skoufos et al., 2021).

Based on the above databases, various computational models have been proposed in recent years, to infer possible associations between microbes and diseases, which can be roughly divided into four categories depending on the technical tools they used, such as the network/graph-based methods, the matrix decomposition-based methods, the conventional machine learning methods, and the deep learning-based methods. Among them, the network/graph-based methods tend to analyze the likelihood of possible microbe–disease associations according to the topological and attribute features of nodes in a heterogeneous network, or a graph is constructed based on known associations between microbes and diseases. For instance, Chen et al. proposed a prediction model named KATZHMDA in 2017 (Chen et al., 2017), which translated the challenge of predicting potential microbe–disease associations into calculating the similarity between nodes based on the length and number of paths linking them in a heterogeneous network. Different from the above network/graph-based methods, the approaches are based on matrix decomposition concentrate on optimizing the product of two potential information matrices to approximate an association matrix with various constraints. For example, Shen et al. designed an identification model CMFHMDA based on collaboration matrix decomposition (Xu et al., 2022). Peng L. et al. proposed a prediction model LDA-VGHB based on singular value decomposition and variational graph autoencoder (Peng et al., 2024a). In addition, traditional machine learning-based approaches focus on training efficient classifiers to detect latent microbe–disease associations based on known associations between microbes and diseases. For instance, Wang et al. introduced a detection model called LRLSHMDA, in which topological information of known microbe–disease pairs was combined with the Laplace regularized least square to build two objective functions and trained an ideal classifier to infer possible disease-associated microbes (Wang et al., 2017). Finally, deep learning-based prediction models aim to discover possible relationships between diseases and microorganisms by developing different deep learning frameworks. For example, Long et al. designed a predictive model to detect latent associations between diseases and microbes by adopting a double-interaction aggregator to improve the representation and aggregation of similar neighborhoods (Long et al., 2021). Moreover, in 2020, Long et al. also proposed a calculative model based on graph attention networks (Veličković et al., 2017), to infer possible human microbe–drug associations (Long et al., 2020). In addition, L. Dayun et al. recommended a computational model MGATMDA to infer possible microbe–disease associations via a multi-component graph attention network (Dayun et al., 2021). In 2023, Peng L et al. proposed a network model based on tree augmentation algorithm and classifier to calculate mediation between ligand receptors (Peng et al., 2024c) and joint scoring based on integrated deep learning and single-cell transcriptomic data, to decrypt ligand receptor-mediated cell-to-cell communication (Peng et al., 2023). In addition, they also devised a bidirectional intentional network named BINDTI based on the attention mechanism, to recognize drug–target interactions in 2024. In the same year, they proposed another dual-net neural architecture and deep neural network to recognize lncRNA-disease association (Peng et al., 2024b). Jiang et al. presented an ensemble approach named SAEROF for large-scale drug–disease association prediction through incorporating the rotation forest and the deep neural network of sparse autoencoder (Jiang et al., 2020). L. Guanghui et al. developed a node-adaptive graph transformer with structural encoding, to predict the association between lncRNA and diseases (Li et al., 2024).

Most of the above methods take multiple features of nodes into account and input them into the same model for learning, ignoring the fact that different models are suitable for learning different types of features. In this study, we introduced two different features such as the attribute features and the topological features of diseases and microbes, respectively, and the difference between these two features is that topological features focus on the spatial relationship and connection in the newly constructed microbe–disease network, emphasizing the structural nature of disease and microbe nodes, whereas the attribute features focus on the attributes and feature vectors of diseases and microbes, describing the specific characteristics of diseases and microbes. To extract these two types of features for diseases and microbes, we designed an improved graph convolutional network (GCAN) and the multi-channel convolutional autoencoder (CSAE) separately. Among them, in GCAN, different from traditional GCNs, we designed an improved transfer matrix, which can aggregate the neighbor information between node pairs, spatialize the constructed heterogeneous network, and extract the relationship between nodes in the space more efficiently. Moreover, during the training process of GCAN, we extracted the features in the form of topological graphs so that we can better obtain the potential topological features in the heterogeneous network. The model CSAE extracts the attribute features of microorganisms mainly through the convolutional and linear layers, and the more important features of the drug itself are more focused on the data itself and can better extract the attribute features of both. In summary, considering that GCAN can propagate information from local neighbors to learn effective representations and has been widely and successfully used in the field of association prediction, we chose GCAN to learn the topological features, while CSAE is selected to learn the attribute features, since CSAE can extract relatively sparse and useful features by introducing a sparsity penalty term on the autoencoder. By using these two different models, we can combine the topological features of spatial associations with the attribute features in the actual data, to more comprehensively assess and predict the association between microbe–disease pairs.

In this article, we improve the transfer matrix for GCN (Kipf and Welling, 2016). The transfer matrix and weighted coefficient are generally used for feature learning in graph propagation neural networks. GCN (Kipf and Welling, 2016) and GAT (Veličković et al., 2017) can be regarded as a special case in graph diffusion-based models using the first-order power of transition matrix. Many graph neural network models such as TAGCN (Du et al., 2017), MixHop (Abu-El-Haija et al., 2019), and DAGNN (Liu et al., 2020) use symmetrically normalized adjacency matrix in GCN as transition matrix. DAGN (Wang et al., 2020) uses attention matrix as transition matrix. PAN (Ma et al., 2019) uses the transition matrix of maximal entropy random walks. Two popular weighting coefficients are personalized PageRank (PPR) (Page et al., 1999; Klicpera et al., 2018) and the heat kernel (Kondor and Lafferty, 2002; Xu et al., 2020), following the previous that more distant neighboring nodes have less influences. PPNP (Klicpera et al., 2018) acts as a post-processing method to propagate output probability generated by an arbitrary model in the graph with PPR. GDC (Klicpera et al., 2019) works as a preprocessing method to recover meaningful neighborhoods from noisy graphs. GraphHeat (Xu et al., 2020) uses the heat kernels as weighting coefficients. Attention walk (Abu-El-Haija et al., 2018) jointly optimizes the node embeddings and weighting coefficients θk. However, the numeric form of weighting coefficients is invariant for each node, which is not flexible. Additionally, some of them are just pre-processing or post-processing methods, which somehow limits their usages. In this study, we used the combination of attention matrix and normalized adjacency matrix as the transition matrix of GCAN. In addition, the GCN layer consists of two parts such as the neighborhood aggregation module and the linear transformation module, which acts as a first-order spectral low-pass type filter because of the addition of self-loops to the re-normalization trick that precedes the symmetric normalization of the transfer matrix. The aggregation operation can be viewed as a matrix multiplication between the weighted adjacency matrix and the node identity matrix. The weighted adjacency matrix is the symmetric normalized adjacency matrix in the GCN. In GAT, the weighted adjacency matrix is the attention matrix with attention scores as entries, which are calculated with representation vectors of directly connected nodes. The improved transfer matrix combines the features of the transfer matrices of the above two models and better combines the attention between the nodes and the neighbor node correlation for feature propagation, and its advantages include GCN that uses the normalized adjacency matrix as a transfer matrix, our weighting matrix is learnable and more flexible, and the predicted results were better at the same time.

Therefore, we use GCAN and CSAE to design a new prediction model GCANCAE. In GCANCAE, a heterogeneous network is constructed by combining the Gaussian interaction profile (GIP) similarity of microorganisms and diseases with the Hamming interaction profile (HIP) similarity of microorganisms and diseases. Then, we introduce GCAN and CSAE to learn the unique topological and attribute representations of microbial and disease nodes in a heterogeneous network, respectively. Later, node heterogeneous networks with different feature matrices obtain the final prediction scores for potential microbial disease associations by integrating these two representations with various microbial and disease features, such as disease functional similarity and microbial functional similarity. Finally, intensive comparative experiments and case studies were conducted to validate the predictive performance of GCANCAE based on HMDAD and Disbiome separately. As a result, the prediction performance of GCANCAE was demonstrated to be better than that of eight state-of-the-art competing methods, which suggested that GCANCAE can not only achieve satisfactory predictive performance but also serve as a useful tool for latent microbe–disease association prediction in the future.

## 2 Materials and methods

### 2.1 Materials

Considering that these two databases such as the HMDAD and the Disbiome have been widely used in the field of microbe–disease association prediction, most of the existing state-of-the-art methods in the field of microbe–disease association prediction adopted these two databases as the basis for their experiments, which may facilitate the comparison between the GCANCAE and these competitive methods. Hence, in this section, we first downloaded known microbe–disease associations from the HMDAD.^1^ After removing duplicated records, we obtained 450 non-redundant experimentally verified microbe–disease associations between 292 microbes and 39 diseases. In addition, 4,351 non-redundant known microbe–disease associations between 1,052 microbes and 218 diseases were further downloaded from the Disbiome.^2^ As a result, the detailed information of these two newly downloaded datasets is presented in the following Table 1.

**Table 1:** The statistics of datasets downloaded from HMDAD and Disbiome.

Datasets	Microbes	Diseases	Associations
HMDAD	292	39	450
Disbiome	1,052	218	4,351

For simplicity, for any given newly downloaded dataset Ω, let $N_{d}$ and $N_{m}$ denote the numbers of different diseases and microbes in Ω, respectively, and it is obvious that we can construct a $N_{d}*N_{m}$ dimensional microbe–disease association adjacency matrix *A* as follows: if the *i*-th disease has a known association with the *j*-th microbe, then there is $A_{ij}=1$ , otherwise, there is $A_{ij}=0$ .

### 2.2 Methods

**Figure 1:**
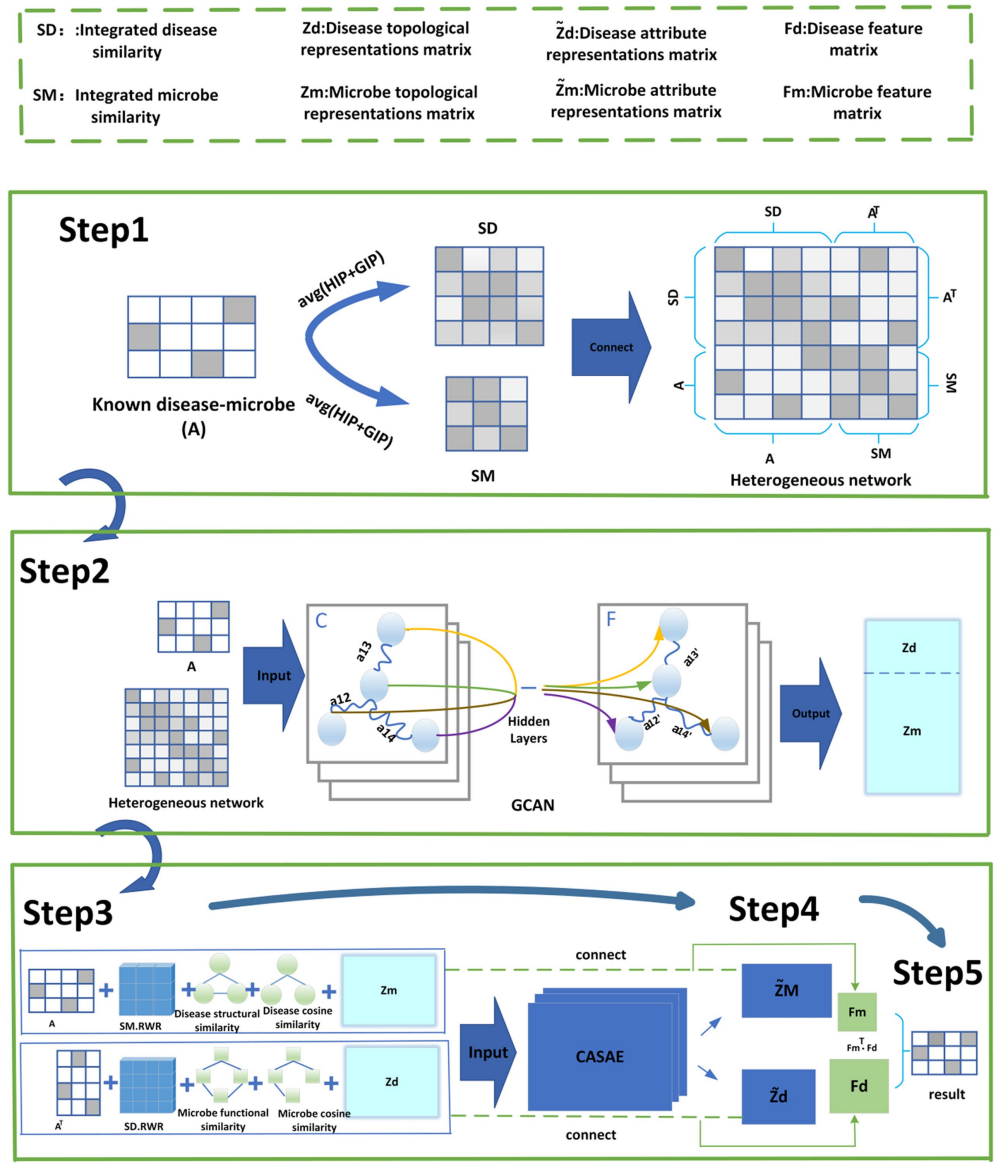
Flowchart of GCANCAE.

As shown in Figure 1, GCANCAE mainly consists of the following five steps:

**Step1**: Constructing a heterogeneous network HN based on multiple similarity metrics of microorganisms and diseases.
**Step2**: Introducing an improved GCN model to extract topological feature representations for microbial and disease nodes in *HN*.
**Step3**: Adopting the CSAE model to capture attribute feature representations for microbial and disease nodes in *HN* separately.
**Step4**: After combining the above two types of feature representations with multiple original features of microbes and diseases, we will construct two integrated feature matrices for diseases and microorganisms, respectively.
**Step5**: Predicted scores for potential microbe–disease associations will be obtained based on the above two feature representations of microbes and diseases.

#### 2.2.1 Construction of the heterogeneous network *HN*

In this section, we will construct a heterogeneous network *HN* by combining the adjacency matrix *A* with multiple similarity measures of microbes and diseases, including the Hamming similarity and the Gaussian Interaction Profile (GIP) kernel similarity as follows:

First, let $\;A\left(m_{i}\right)$ and $A\left(m_{j}\right)$ represent the *i-th* column and the *j-th* column of A separately, and then for any two given microbes $m_{i}$ and $m_{j}$ , we will estimate the GIP kernel similarity $GM\left(i,j\right)\in R^{N_{m}*N_{m}}$ between these two microbes by the following Equations (1, 2):


(1) $$GM\left(i,j\right)=\exp\left(-\gamma_{m}||A\left(m_{i}\right)-A\left(m_{j}\right)|{|}^{2}\right)$$

(2) $$\gamma_{m}=\frac{N_{m}}{\sum_{i=1}^{N_{m}}||\;A\left(m_{i}\right)\;|{|}^{2}}$$

Here, || · || is the Frobenius norm.

In addition, inspired by the study proposed by Xu et al. (2021), for any two given microbes $m_{i}$ and $m_{j}$ , the Hamming similarity between them can be calculated according to the Equation (3):


(3) $$HM\left(i,j\right)=1-\frac{\sum_{k=1}^{N_{m}}\left|A\left(k,i\right)-A(k,j)\right|}{N_{m}}$$

Next, in a similar way, let $A\left(d_{i}\right)$ and $A\left(d_{j}\right)$ denote the *i-th* row and the *j-th* row of A , respectively, and then for any two given diseases $d_{i}$ and $d_{j}$ , we can obtain the GIP kernel similarity between them by the following equations (4, 5):


(4) $$GD\left(i,j\right)=\exp\left(-\gamma_{d}||\;A\left(d_{i}\right)-A\left(d_{j}\right)\;|{|}^{2}\right)$$

(5) $$\gamma_{d}=\frac{N_{d}}{\sum_{i=1}^{N_{d}}||\;A\left(d_{i}\right)\;|{|}^{2}}$$

Furthermore, it was obvious that we can also obtain the Hamming similarity between $d_{i}$ and $d_{j}$ according to the Equation (6):


(6) $$HD\left(i,j\right)=1-\frac{\sum_{k=1}^{N_{d}}\left|A\left(i,k\right)-A(j,k)\right|}{N_{d}}$$

Thus, it is easy to observe that we can synthesize an integrated microbe similarity matrix $SM\in R^{N_{m}*N_{m}}$ and an integrated disease similarity matrix $SD\in R^{N_{d}*N_{d}}$ through combining the GIP kernel similarity matrix and the HIP similarity matrix of microbe or diseases separately according to the following Equations (7, 8):


(7) $$SM=\frac{GM+HM}{2}$$

(8) $$SD=\frac{GD+HD}{2}$$

Finally, based on the above newly obtained matrices $SM\in R^{N_{m}*N_{m}}$ and $SD\in R^{N_{d}*N_{d}}$ , it is obvious that we can construct a heterogeneous network $HN\in R^{\left(N_{d}+N_{m}\right)*\left(N_{d}+N_{m}\right)}$ based on the Equation (9):


(9) $$HN=\left[\begin{array}{cc} SD & A\\ A^{T} & SM \end{array}\right]$$

#### 2.2.2 Extraction of topological feature representations for nodes in HN via GCN

**Figure 2:**
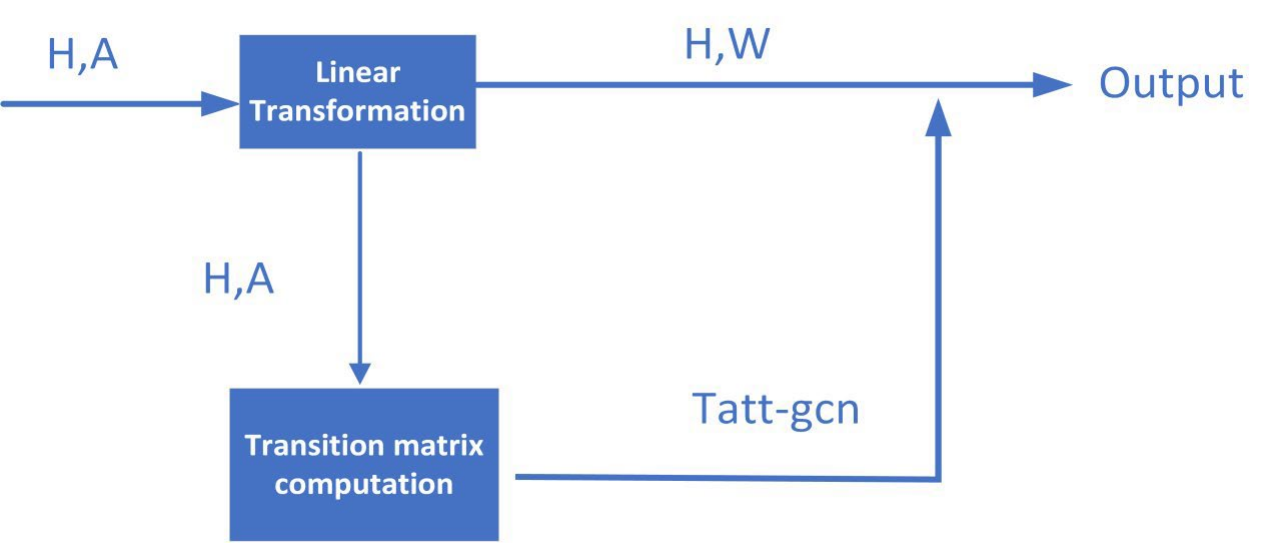
Flowchart of the improved GCN.

In this section, inspired by the idea proposed by Sun and Wu (2020), in order to better extract the topological feature representations for nodes in the heterogeneous network HN , as shown in Figure 2, we will first design an improved transition matrix of GCN according to the Equation (10):


(10) $$T_{att-gcn}={\left(IN+D\right)}^{-\frac{1}{2}}\;\;D_{att,r}-1A_{att}{\left(IN+D\right)}^{-\frac{1}{2}}$$

where *IN* $\in R^{\left(N_{d}+N_{m}\right)*\left(N_{d}+N_{m}\right)}$ is a $\left(N_{d}+N_{m}\right)*\left(N_{d}+N_{m}\right)$ dimensional identity matrix, and the matrices $D_{att,r}$ , $A_{att}$ , and *D* are defined by the following Equations (11-13) respectively:


(11) $${\left[A_{att}\right]}_{ij=}\left\{\begin{array}{c} \exp\left(LeakyRelu\left(\left[Hi||Hj\right]\cdot a\right)\right),j\in Ni\cup \left\{i\right\}\\ 0\;,j\notin Ni\cup \left\{i\right\} \end{array}\right.$$

(12) $${\left[D_{att,r}\right]}_{ij}=\underset{j\in ℵi\;}{\sum }\exp\left(LeakyRelu\left(\left[Hi||Hj\right]\cdot a\right)\right)$$

(13) $$D_{\left(N_{d}+N_{m}\right)\left(N_{d}+N_{m}\right)}=\left[\begin{array}{cc} \overset{N_{m}}{\underset{j=1}{\sum }}A_{ij} & zero\left(N_{d}\times N_{d}\right)\\ zero\left(N_{m}\times N_{m}\right) & \overset{N_{d}}{\underset{i=1}{\sum }}A_{ij} \end{array}\right]$$

In the above equations, zero(k×k) is a k×k dimensional zero matrix, *IN + D* means the degree matrix after adding the self-loop, Ni is the set of adjacent nodes of the *i*-th node in *HN*, “.” denotes the inner product, “||” indicates the connection operation, *Hi* means the representation vector of the *i*-th node in *HN*, and *a* is the attention vector.

Next, we will adopt the transition matrix *T_att-gcn_*, to participate in the layer-by-layer propagation of GCN according to the Equation (14):


(14) $$H^{\left(l+1\right)}=\sigma \left(T_{att-gcn}H^{\left(l\right)}W^{\left(l\right)}\right)$$

where σ is the activation function, *l* denotes the number of layers in GCN, $W^{\left(l\right)}$ indicates the trainable weights of the *l*-th layer in GCN, and $H^{\left(l\right)}$ represents the input of the *l*-th layer in GCN. In this study, we will take the heterogeneous network *HN* as the original input $H^{\left(0\right)}$ .

Obviously, based on the above newly constructed GCN, we can easily obtain a new output matrix $Z=\left[\begin{array}{c} Z^{d}\\ Z^{m} \end{array}\right]\in R^{\left(N_{d}+N_{m}\right)*l}$ , where $Z^{d}\in R^{N_{d}*l}$ and $Z^{m}\in R^{N_{m}*l}$ represent the disease and microbial features newly extracted by GCN, respectively.

Moreover, based on the above newly-obtained matrix *Z*, we will further design a decoder based on the Equation (15):


(15) $$ZZ=sigmoid\left(Z\cdot Z^{T}\right)$$

After that, we will adopt the MSE loss function to calculate the mean of the sum of squares of the differences between *ZZ* and the *HN* based on the Equation (16):


(16) $$L_{MSE}=\frac{1}{N_{d}+N_{m}}\overset{N_{d}+N_{m}}{\underset{i=1}{\sum }}||\;ZZ\left(i\right)-HN\left(i\right)\;|{|}^{2}$$

where ZZ(i) and HN(i) denote the *i*-th row of ZZ and HN , respectively.

Finally, we will select the Adam optimizer (Kingma and Adam, 2014) to optimize the predicted results and apply the final trained $Z^{d}$ and $Z^{m}$ to future prediction tasks.

#### 2.2.3 Extraction of attribute representations for nodes in *HN* via CSAE

In this section, we will further adopt the Random Walk with Restart (*RWR*) (Köhler et al., 2008), cosine similarity, and functional similarity, to obtain the local and global intrinsic attribute features of nodes in *HN* efficiently.

First, we will apply *RWR* on *SM* and *SD* to discover the correlation and importance between nodes in *HN* according to the Equation (17):


(17) $$\;q_{i}^{t+1}=\varphi Mq_{i}^{t}+\left(1-\varphi \right)\epsilon_{i}\;$$

where φ is the restart probability and will be set to 0.1 according to traditional experimental result (Tan et al., 2022) and $q_{i}^{t}$ is a vector in which the *i*-th element holds the probability of being at the node i during the t-th time slot. *M* denotes the transfer probability matrix and $\epsilon_{i}\in R^{1*m}$ is the initial probability vector of node i, which is defined by the Equation (18):


(18) $$\epsilon_{ij}=\{\begin{array}{c} 1\;\;if\;i=j\\ 0otherwise \end{array}$$

Obviously, based on the above equations, after applying *RWR* on *SM* and *SD* separately, we can obtain a novel $N_{d}*N_{d}$ dimensional matrix $SD^{MM}$ and a new $N_{m}*N_{m}$ dimensional matrix $SM^{DD}$ successively.

Next, for any two given disease nodes $d_{i}$ and $d_{j}$ in *HN*, we will calculate the cosine similarity between them according to the Equation (19):


(19) $$S_{D}^{COS}\left(i,j\right)=COS\left(A\left(i,:\right),A\left(j,:\right)\right)=\frac{A\left(i,:\right)A^{T}\left(j,:\right)}{\left||\;A\left(i,:\right)\;||\times ||\;A\left(j,:\right)\;|\right|}$$

Moreover, in the similar way, for any two given microbe nodes $m_{i}$ and $m_{j}$ in *HN*, we will calculate the cosine similarity between them by the Equation (20):


(20) $$S_{M}^{COS}\left(i,j\right)=COS\left(A\left(:,i\right),A\left(:,j\right)\right)=\frac{A\left(:,i\right)A^{T}\left(:,j\right)}{\left||\;A\left(:,i\right)\;||\times ||\;A\left(:,j\right)\;|\right|}$$

Obviously, based on the above equations, we can obtain two matrices $S_{D}^{COS}\in R^{N_{d}*N_{d}}$ and $S_{M}^{COS}\in R^{N_{m}*N_{m}}$ simultaneously.

**Figure 3:**
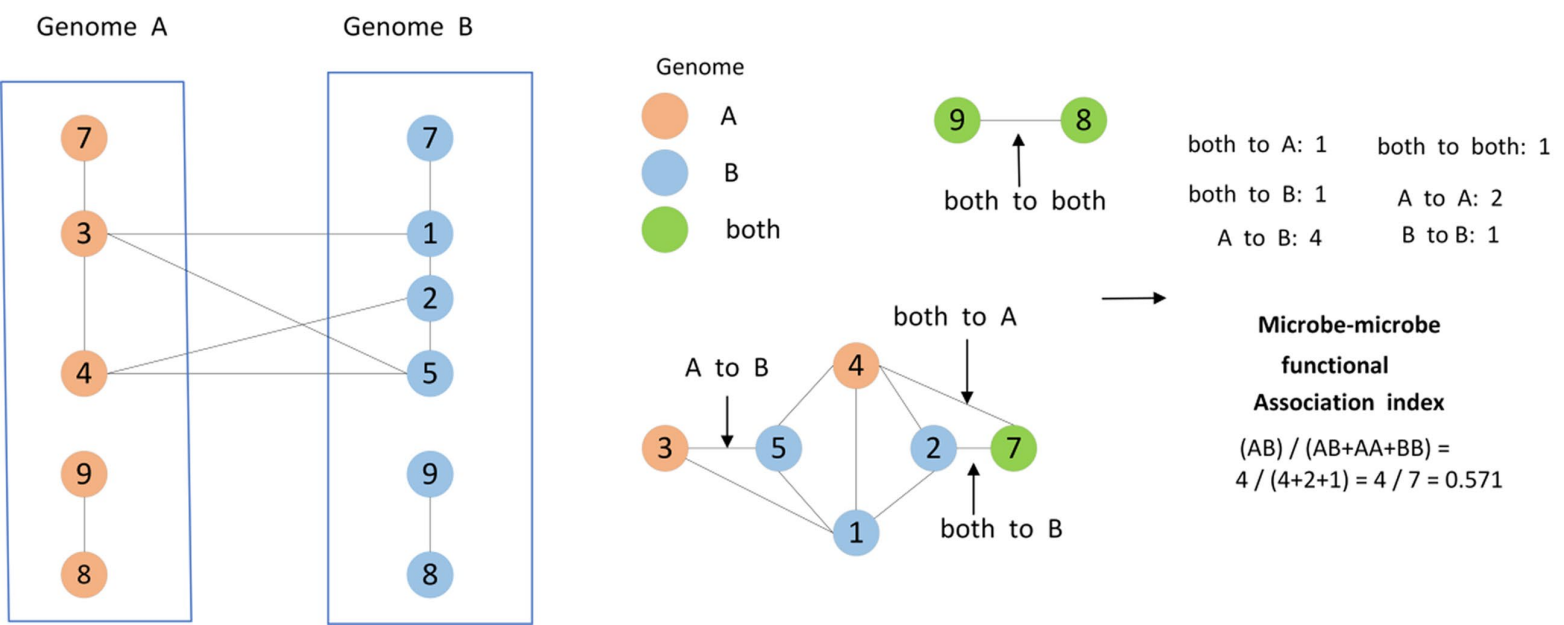
Two microbial species A and B were defined, containing five and six gene families, respectively, while two gene families occur only in A (3, 4), three gene families occur only in B (1, 2, 5), and three gene families occur in both species A and species B (7, 8, 9). These three types of gene families mark the nodes of the protein functional association network. Moreover, the edges connecting the gene families were categorized into six classes, namely, both to A, both to A and B, both to B, A to A, B to B, A to B. As shown in the figure, the similarity of different edges was calculated by counting the number of different edges.

Furthermore, based on the method proposed by Kamneva OK (Kamneva, 2017), as shown in Figure 3, for any two given microbes $m_{i}$ and $m_{j}$ , we will calculate the functional similarity between them as well, and as a result, we can obtain a novel $N_{m}*N_{m}$ dimensional microbe functional similarity matrix $S^{MFS}\in R^{N_{m}*N_{m}}$ based on these $N_{m}$ different newly downloaded microbes in *Ω*.

After that, based on the assumption that functionally similar diseases tend to be in contact with functionally similar genes (Xu and Li, 2006), in this method, Human PPI datasets were downloaded from the Online Predicted Human Interaction Database (OPHID) (Brown and Jurisica, 2005) that is used to establish the PPI network. The resulting features are input into the KNN classifier to obtain the disease functional similarity. We found the functional similarity between the corresponding diseases to establish the disease similarity matrix. After that, we can obtain a new $N_{d}*N_{d}$ dimensional disease functional similarity matrix $S^{DFS}\in R^{N_{d}*N_{d}}$ based on these $N_{d}$ different newly downloaded diseases in *Ω*.

Obviously, based on the above newly obtained matrices A , $SD^{MM}$ , $S_{D}^{COS}$ and $S^{DFS}$ , we can finally construct a new disease attribute matrix $A^{D}$ based on the Equation (21):


(21) $$A^{D}=\left[A;SD^{MM};S_{D}^{COS};S^{DFS}\right]$$

In a similar way, based on the above newly obtained matrices A , $SM^{DD}$ , $S_{M}^{COS}$ and $S^{MFS}$ , we can also construct a new microbe attribute matrix $A^{M}$ based on the Equation (22):


(22) $$A^{M}=\left[A^{T};SM^{DD};S_{M}^{COS};S^{MFS}\right]$$

**Figure 4:**
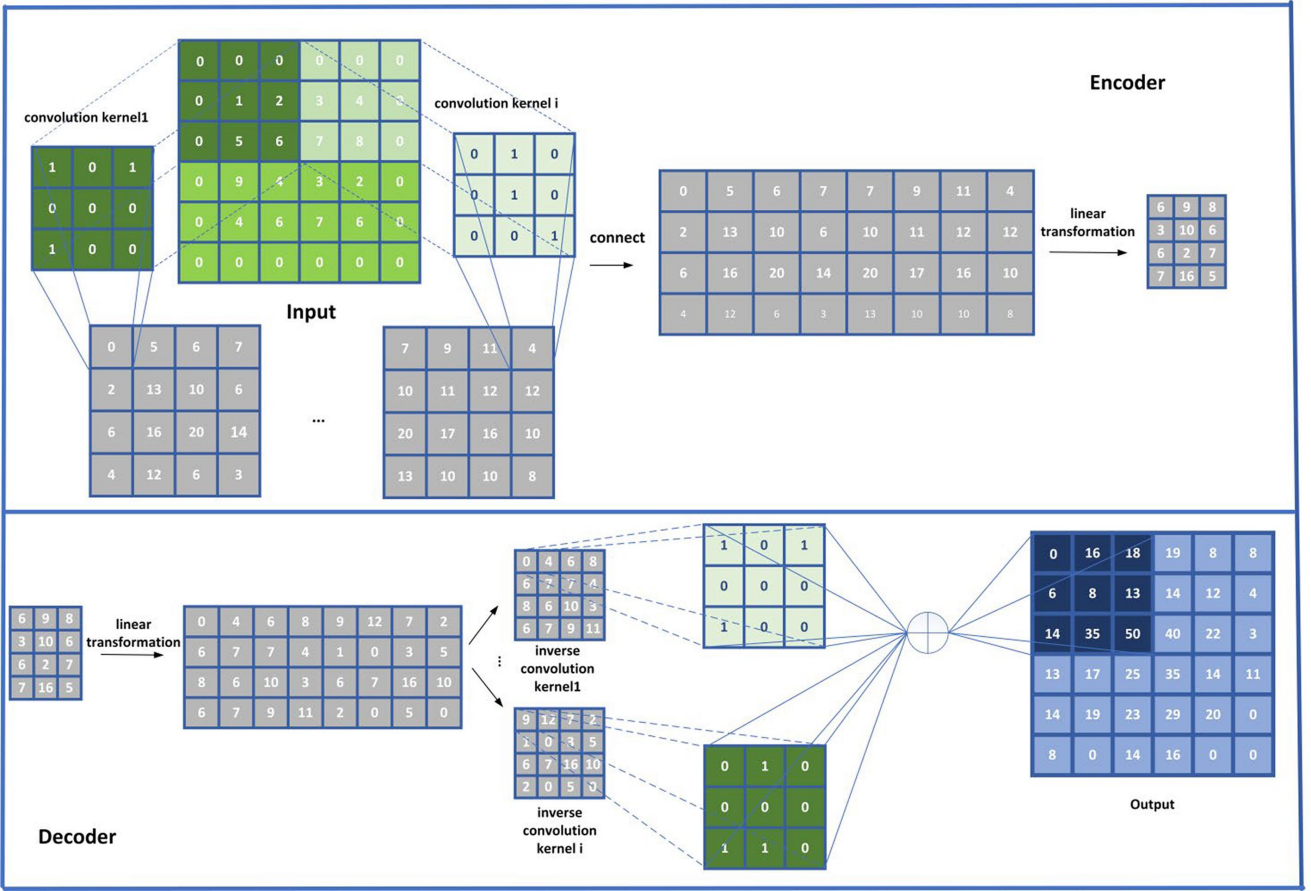
Flowchart of the CSAE.

Based on the above two matrices $A^{D}$ and $A^{M}$ , in order to extract more important attribute representations for disease and microbial nodes in *HN*, as shown in Figure 4, we will input $A^{D}$ and $A^{M}$ to the CSAE separately according to the following steps:

Step 1 (Convolutional Encoder): First, in order to realize the convolutional coding, we will input $A^{D}$ and $A^{M}$ to the CSAE, respectively, based on the Equation (23):


(23) $$f_{X}=Relu\left(A^{X}⊗W+b_{encoder}\right)$$

where $A^{X}\in$ { $A^{D},A^{M}$ } represents the input of the CSAE, *“* ⊗ *"*indicates a convolutional operation, and W denotes the convolution kernel used for each channel. In this study, we will set the convolution kernel size to 3 * 3. In addition, $b_{encoder}$ represents the offset, Relu() means the activation function. Hence, it is easy to know that there is $f_{X}$ $\in R^{N_{r}*N_{c}*l}$ , where $N_{r}$ and $N_{c}\;$ denote the lengths of the rows and columns in the input matrix $A^{X}$ , respectively, and *l* represents the number of convolution kernels in the CSAE.

Step 2 (Linear Encoder): In this step, the $f_{t}$ will be performed the linear sparse coding on after dimensionality reduction splicing of the $f_{X}$ based on the Equations (24, 25):


(24) $$f_{t}=textflatten\left(f_{X}\right)$$

(25) $$h_{W,B}=\sigma \left(W_{encoder}*f_{t}+B_{encoder}\right)$$

Where textflatten() is the function used to flatten the matrix $f_{X}$ to a two-dimensional vector $f_{t}$ ∈ $R^{N_{r}*\left(N_{c}*l\right)}$ . Besides, σ() is the activation function, $W_{encoder}$ represents the encoding weight, $B_{encoder}$ denotes the bias term, and $h_{W,B}$ means the intermediate hidden layer.

Step 3 (Linear Decoder): In this step, $\;h_{W,B}$ will be decoded linearly based on the Equation (26):


(26) $$y_{W,B}=\sigma \left(W_{decoder}h_{W,B}+B_{decoder}\right)$$

where $W_{decoder}$ indicates that the weight $B_{decoder}$ at decoding is the decoding bias term.

Step 4 (Convolutional Decoder): In this step, based on the newly obtained $y_{W,B}\in R^{N_{r}*\left(N_{c}*l\right)}$ , we will construct a multi-channel feature matrix $f\in R^{N_{r}*N_{c}*l}$ first, and then, we input it to the deconvolution layer for multi-channel convolution decoding based on the Equations (27, 28):


(27) $$f=\left[y_{W,B}\_1,y_{W,B}\_2,\ldots ,y_{W,B}\_l\right]$$

(28) $$F=Relu\left(f◉W+b_{\mathrm{decoder}}\right)$$

where $y_{W,B}\_i\in R^{N_{r}*N_{c}}$ denotes the *i*-th channel of the matrix $y_{W,B}$ obtained by transversal partitioning, “ [] ” Indicates the splice operation, “◉” represents the deconvolution operation, and W represents the convolution kernel in the deconvolution layer. In this study, we will set the convolution kernel size to 3 * 3. In addition, $b_{decoder}$ represents the offset, Relu() indicates the activation function, and *l* represents the number of convolution kernels in the CSAE.

Obviously, based on the above Eq. 27, we can first obtain a 3D tensor $f\in R^{Nr*Nc*l}$ by partitioning the original 2D matrix $y_{W,B}$ to *l* channels according to the transversal dimension of the $y_{W,B}$ , and then, based on the above Eq. 28, the decoded feature representation *F* of nodes in *HN* can be obtained.

Step 5: In this step, in order to ensure the sparsity of the hidden layer, we will further add the following penalty items defined in the Equation (29) to the CSAE as well:


(29) $$P_{penalty}=\overset{S_{2}}{\underset{t=1}{\sum }}KL(\rho \;||\;{\hat{\rho }}_{t})$$

where $S_{2}$ represents the number of hidden layer neurons in the CSAE, $\hat{\rho_{t}}$ stands for the average activity of a hidden neuron t , and $KL(\rho \;||\;{\hat{\rho }}_{t})$ denotes the relative entropy between two Bernoulli random variables with means of ρ and $\hat{\rho_{t}}$ respectively, which is defined by the Equation (30):


(30) $$KL(\rho \;||\;{\hat{\rho }}_{t})=\rho log\frac{\rho }{{\hat{\rho }}_{t}}+\left(1-\rho \right)\log\frac{1-\rho }{1-{\hat{\rho }}_{t}}$$

Based on the above steps, it is obvious that we can obtain two output matrices $A^{DD}$ and $A^{MM}$ after inputting $A^{D}$ and $A^{M}$ to the CSAE separately.

Step 6: Finally, similar to the implementation of GCAN, we will also utilize the Adam optimizer and MSE loss function for the optimization of the CSAE. Using the disease attribute representation as an example, the sparse penalty terms will be introduced into the loss function throughout the optimization phase according to the Equation (31):


(31) $$L_{sparse}=\frac{1}{N_{d}}\overset{N_{d}}{\underset{k=1}{\sum }}||\;A^{DD}\left(k\right)-A^{D}\left(k\right)|{|}^{2}+\beta P_{penalty}$$

Here, *β* is the weight of the sparse penalty and will be set to 0.1 in this manuscript. $A^{DD}\left(k\right)$ and $A^{D}\left(k\right)$ denote the *k*-th row of $A^{DD}$ and $A^{D}$ , respectively.

According to the above steps, it is easy to observe that a low-dimensional drug attribute representation matrix ${\tilde{A}}^{D}\in R^{N_{d}*k}$ and a low-dimensional microbe attribute representation matrix ${\tilde{A}}^{M}\in R^{N_{m}*k}$ can be obtained simultaneously by adopting the CSAE after it has been well trained.

#### 2.2.4 Construction of the eigenmatrix of disease and microbe

Inspired by the method proposed by Xuan et al. (2021), in this study, we first spliced the functional similarity and the cosine similarity to maintain the original attributes of the nodes. Then, we combined the random wandering with the topological and attribute features extracted by GCAN and CSAE, to obtain the neighbor information of nodes and the learned new features. Thus, the integrated feature matrix would be more conducive to the prediction of potential microbe–disease associations. Finally, based on the above newly obtained disease-related matrices $Z^{d}$ , ${\tilde{A}}^{D}$ , $S^{DFS}$ , $S_{D}^{COS}$ , $SD^{MM}$ and the adjacency matrix *A*, we can construct a new disease eigenmatrix $F_{D}$ based on the Equation (32):


(32) $$F_{D}=\left[Z^{d};\;{\tilde{A}}^{D};\;S^{DFS};\;A;\;S_{D}^{COS};\;A;\;SD^{MM};\;A\right]$$

In a similar way, by combining the above newly obtained microbe-related matrices $Z^{m}$ , ${\tilde{A}}^{M}$ , $S^{MFS}$ , $S_{M}^{COS}$ , $SM^{DD}$ and the transposed adjacency matrix $A^{T}$ , we can create a novel microbe eigenmatrix $F_{M}$ according to the Equation (33):


(33) $$F_{M}=\left[\;Z^{m};\;{\tilde{A}}^{M};\;A^{T};\;S^{MFS};\;A^{T};\;S_{M}^{COS};\;A^{T};\;SM^{DD}\right]$$

#### 2.2.5 Calculation of the predicted scores

Based on the above two newly constructed eigenmatrices $F_{D}$ and $F_{M}$ , for any given disease $d_{i}$ and microbe $m_{j}$ , it was obvious that we could estimate the possibility of potential association between them by adopting the following inner product according to the Equation (34):


(34) $$S\left(i,j\right)=Sigmoid\left(\;F_{D}\left(d_{i}\right)�F_{M}{\left(m_{j}\right)}^{T}\right)$$

Here, $F_{D}\left(d_{i}\right)$ denotes the *i*-th row of $F_{D}$ , while $F_{M}\left(m_{j}\right)$ denotes the *j*-th row of $F_{M}$ .

## 3 Results

### 3.1 Comparison with advanced methods

In this section, in order to evaluate the prediction performance of GCANCAE, we would compare it with eight different types of cutting-edge microbe–disease association prediction methods, such as KATZHMDA (Chen et al., 2017), which used KATZ to speculate on potential microbe–disease correlations, LRLSHMDA (Wang et al., 2017), which used the Laplacian-based regularized least-squares framework to estimate the possible associations between microbes and diseases, NTSHMDA (Luo and Long, 2020), which adopted the random walk with restart to forecast potential microbe–disease connections, BiRWMP (Wang et al., 2019), which introduced double random walk to forecast microbiological infections, NBLPIHMDA (Fan et al., 2020), which utilized a two-way marker transmission approach to detect probable microbe–disease correlations, HMDA-pred (Li et al., 2021), which adopted the network consensus projection and multi-data integration to identify microbe-related diseases, BPNNHMDA (Cai et al., 2021), which was developed based on backpropagation neural networks to deduce possible correlations between microbes and diseases, and GATMDA (Long et al., 2021), which used a graph attention network with a full inductive matrix to detect associations between microbe and disease pairs.

During experiments, for a fair comparison, we would test all these competing algorithms based on their original optimal parameters. In addition, intensive comparison experiments would be implemented based on two different databases of HMDAD and Disbiome under the *k*-fold cross-validation (CV) framework developed by Cai et al. (2021). In this case, we randomly selected 20% of known associations and 20% of unknown associations as the test set, while we selected the remaining 80% of known and unknown associations as the training set. Then, we implemented the 5-fold CV 10 times to obtain the final prediction results. Based on HMDAD and Disbiome, the final comparison results were shown in the following Tables 2, 3 separately.

**Table 2:** Comparison results of performance between GCANCAE and eight competitive approaches based on the HMDAD database in the 5-fold CV and the 2-fold CV.

Methods	Classification of methods	AUC (5-fold cv)	AUC (2-fold cv)
KATZHMDA	Network or graph based methods	0.8301 **±** 0.0033	0.8171 **±** 0.0051
LRLSHMDA	Traditional machine learning methods	0.8794 **±** 0.0029	0.8595 **±** 0.0056
NTSHMDA	Traditional machine learning methods	0.8896 **±** 0.0038	0.8623 **±** 0.0061
BiRWMP	Traditional machine learning methods	0.8777 **±** 0.0089	0.8698 **±** 0.0079
NBLPIHMDA	Traditional machine learning methods	0.8958 **±** 0.0027	0.8799 **±** 0.0062
HMDA-pred	Network or graph based methods	0.9361 **±** 0.0037	0.9053 **±** 0.0029
BPNNHMDA	Deep learning based methods	0.9127 **±** 0.0009	0.8955 **±** 0.0018
GATMDA	Deep learning based methods	0.9554 **±** 0.0184	0.9538 **±** 0.0049
**GCANCAE**	**Network or graph based methods**	**0.9770** ± **0.0002**	**0.9741** ± **0.0017**

The best predicted values were shown in bold, and the second-best results were underlined.

**Table 3:** Comparison results of performance between GCANCAE and eight competitive approaches based on the Disbiome database in the 5-fold CV and the 2-fold CV.

Methods	Classification of methods	AUC (5-fold cv)	AUC (2-fold cv)
KATZHMDA	Network or graph based methods	0.6779 **±** 0.0141	0.6696 **±** 0.0058
LRLSHMDA	Traditional machine learning methods	0.7356 **±** 0.0236	0.7187 **±** 0.0127
NTSHMDA	Traditional machine learning methods	0.8294 **±** 0.0071	0.8086 **±** 0.0058
BiRWMP	Traditional machine learning methods	0.8344 **±** 0.0089	0.8139 **±** 0.0060
NBLPIHMDA	Traditional machine learning methods	0.8426 **±** 0.0177	0.8275 **±** 0.0099
HMDA-pred	Network or graph based methods	0.8515 **±** 0.0376	0.8367 **±** 0.0384
BPNNHMDA	Deep learning based methods	0.8704 **±** 0.0158	0.8515 **±** 0.0136
GATMDA	Deep learning based methods	0.9307 **±** 0.0079	0.9296 **±** 0.0154
**GCANCAE**	**Network or graph based methods**	**0.9617 ± 0.0120**	**0.9616 ± 0.0001**

The best predicted values are shown in bold, and the second-best results were underlined.

After observing the Table 2, it is easy to observe that GCANCAE can achieve the best predictive performance with an average AUC of 0.9770 ± 0.0002 in the 5-fold CV and 0.9741 ± 0.0017 in the 2-fold CV, respectively, which are superior to that achieved by all these eight competing approaches.

After observing the Table 3, it is obvious that GCANCAE can obtain the best predictive performance with an average AUC of 0.9617 ± 0.0120 in the 5-fold CV and 0.9616 ± 0.0001 in the 2-fold CV separately, which further demonstrates that GCANCAE outperforms all those state-of-the-art prediction models.

### 3.2 Sensitivity analysis of hyperparameters

In GCANCAE, we introduced some hyperparameters, such as the learning rates *lr*1 and *lr*2, the dimensionality *k*1 of the node topological representation, the dimension *k*2 of the node attribute representation, the number of channels *l,* and the number of layers *GCAN_l* of GCAN. In this section, we would determine suitable values for these hyperparameters based on the 5-fold CV and the HMDAD database.

For the hyperparameters *k*1 and *k*2, we compared the experimental results while *k*1 and *k*2 varied from 32, 64, 128 to 256, respectively, and found that GCANCAE could obtain the best performance when *k*1 was set to 128 and *k*2 was set to 32. In addition, for the learning rates *lr*1 and *lr*2, we compared the experimental results, while *lr*1 and *lr*2 varied in the range of 0.001, 0.05, 0.01, and 0.1, respectively. For the channel number *l*, we compared the experimental results, while *l* varied between 3, 6, and 9. On the layers of GCAN, we calculated the values of the model when *GCAN_l* is 1, 2, and 3. It was finally found that GCANCAE could achieve the best AUC values when *lr*1 was set to 0.01, *lr*2 was set to 0.1, channel number *l* was set to 6, and *GCAN_l* was set to 1.

**Figure 5:**
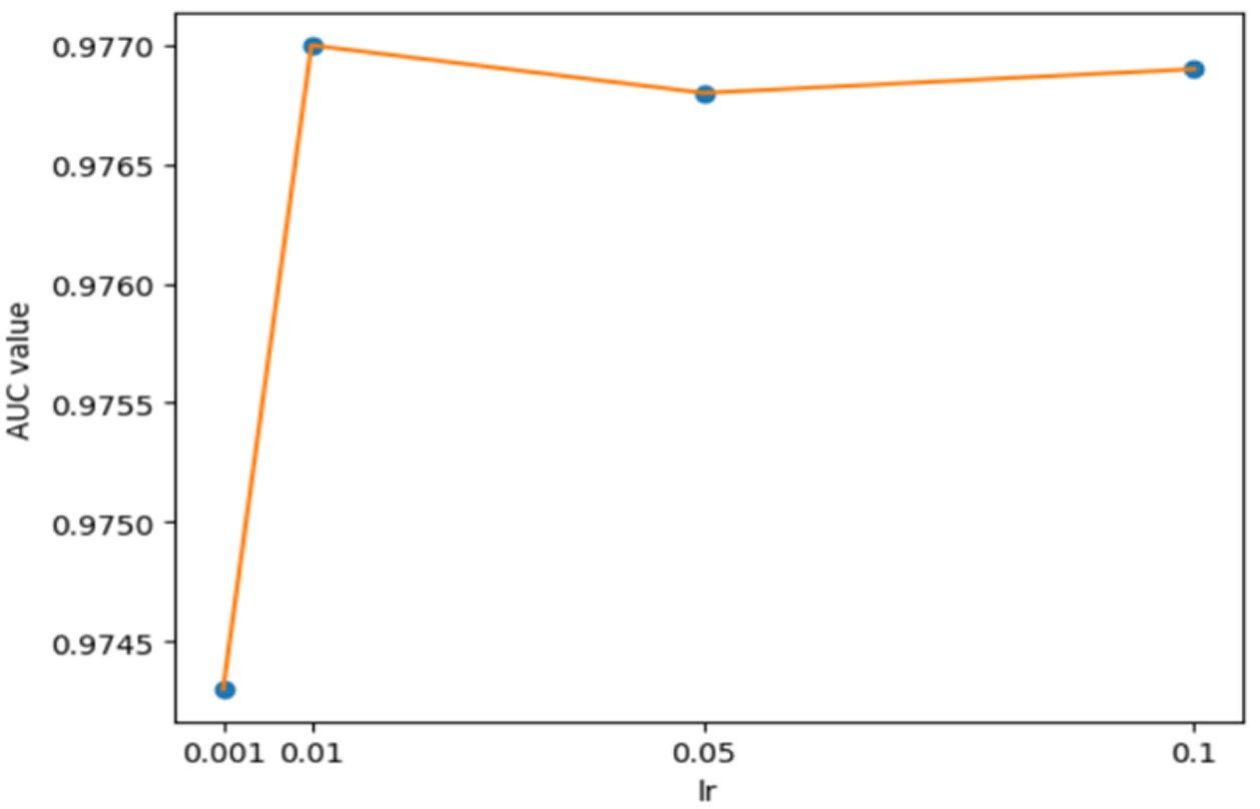
AUCs achieved by GCANCAE with different learning rates *lr*1 (GCAN).

**Figure 6:**
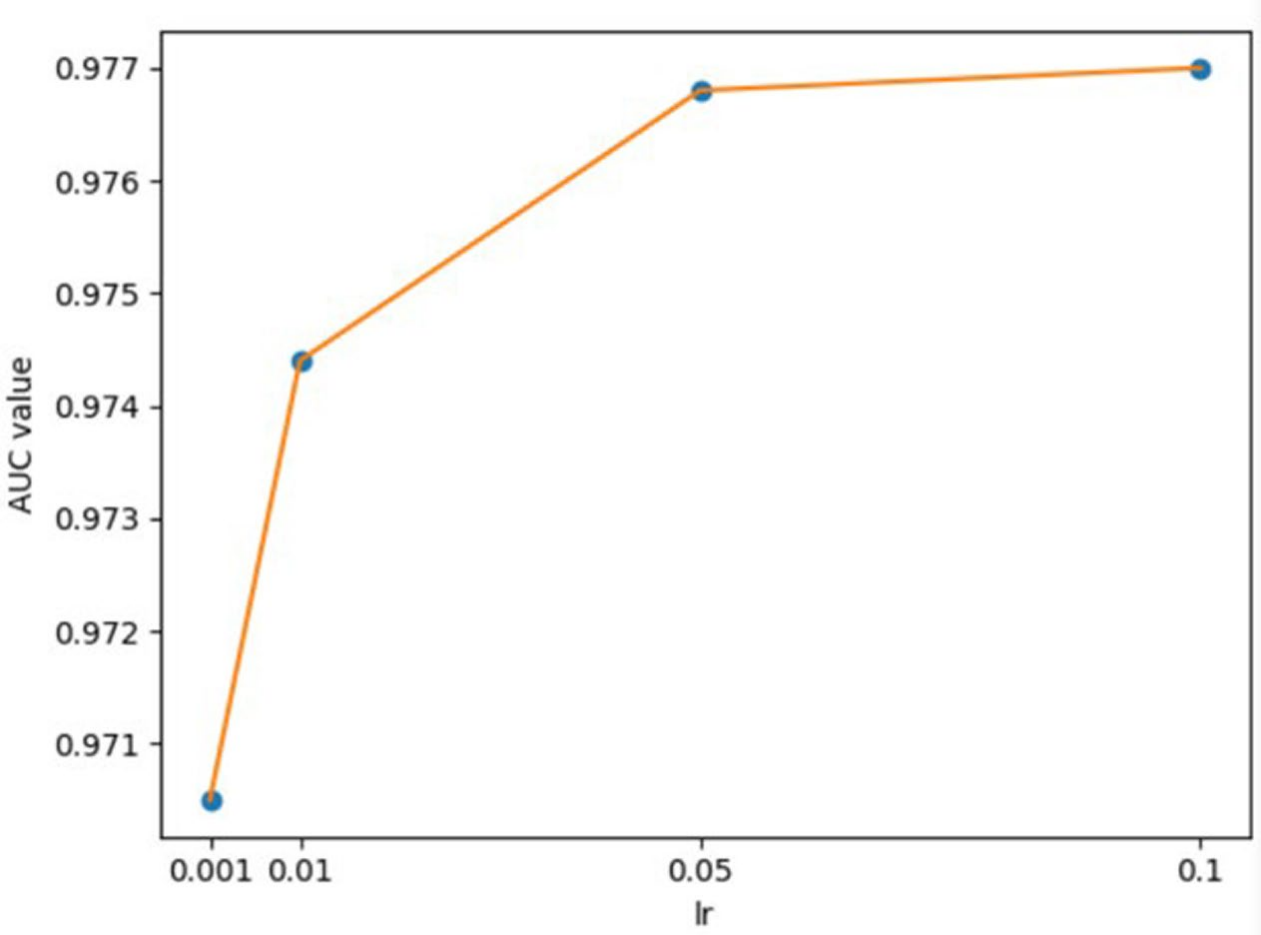
AUCs achieved by GCANCAE with different learning rates *lr*2 (CSAE).

**Figure 7:**
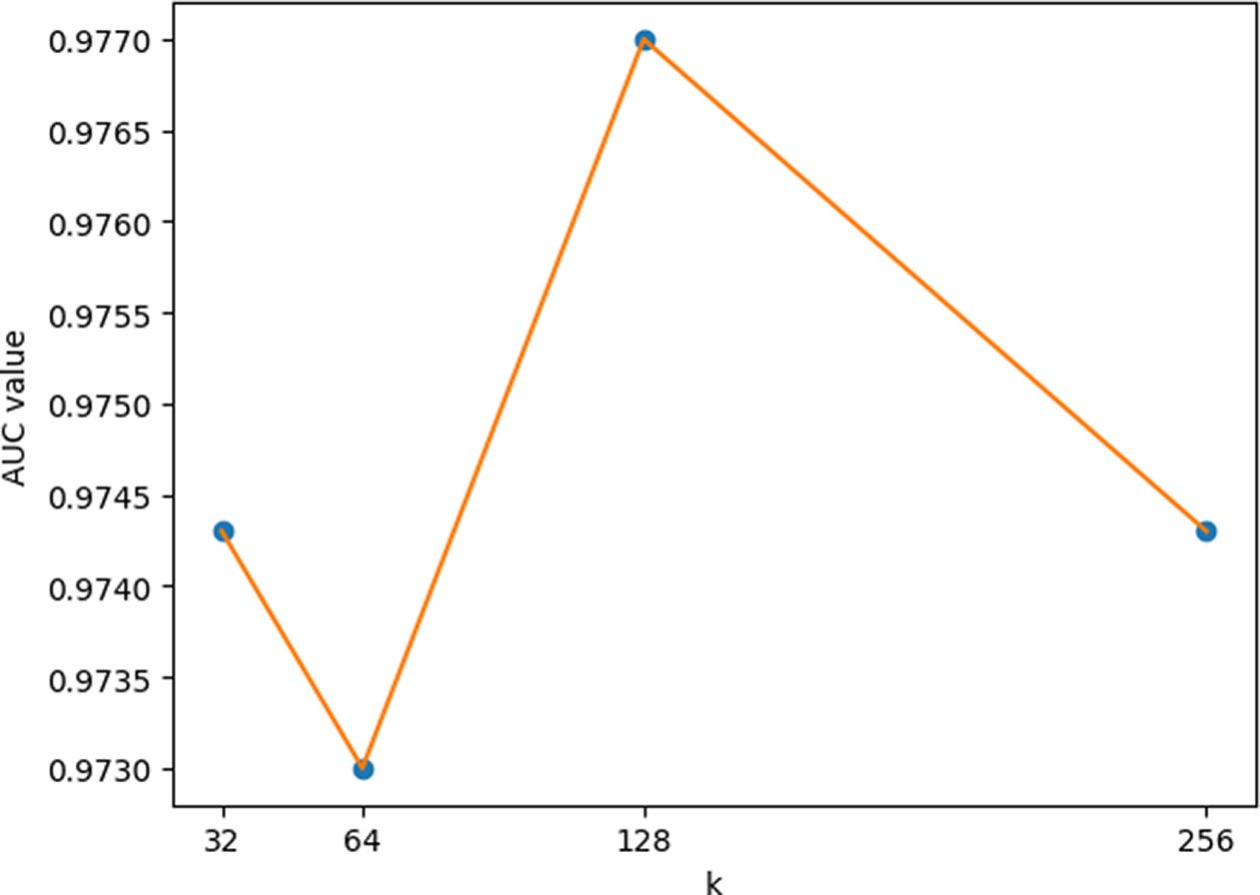
AUCs achieved by GCANCAE with different dimensions of node attribute representation (*k*2).

**Figure 8:**
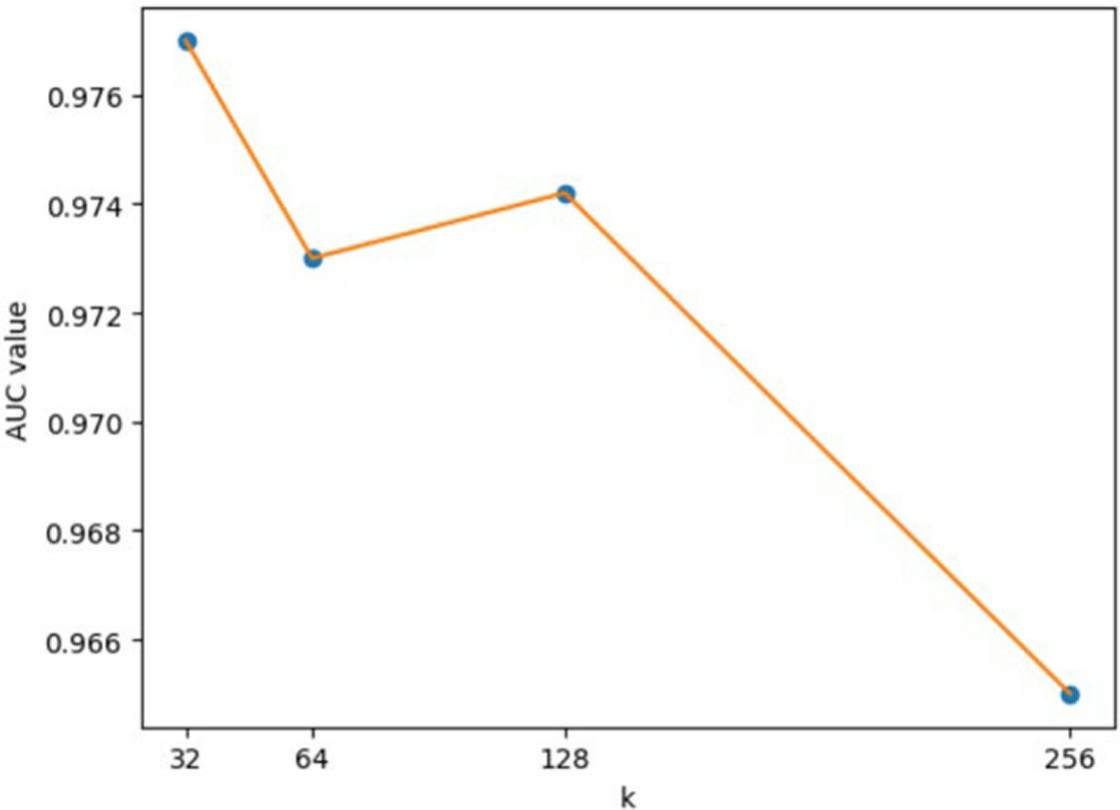
AUCs achieved by GCANCAE with different dimensions of node topological of node topological representation (*k1*).

**Figure 9:**
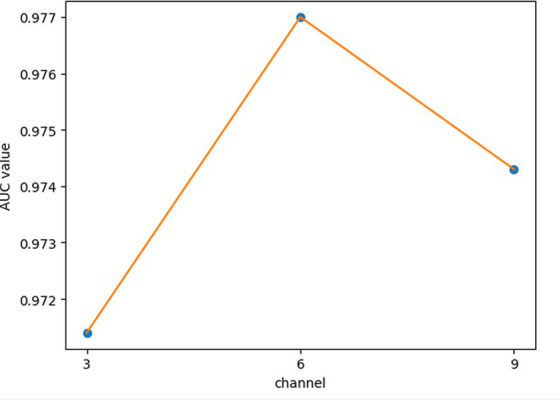
AUCs achieved by GCANCAE with different channels of node attribute representation.

**Figure 10:**
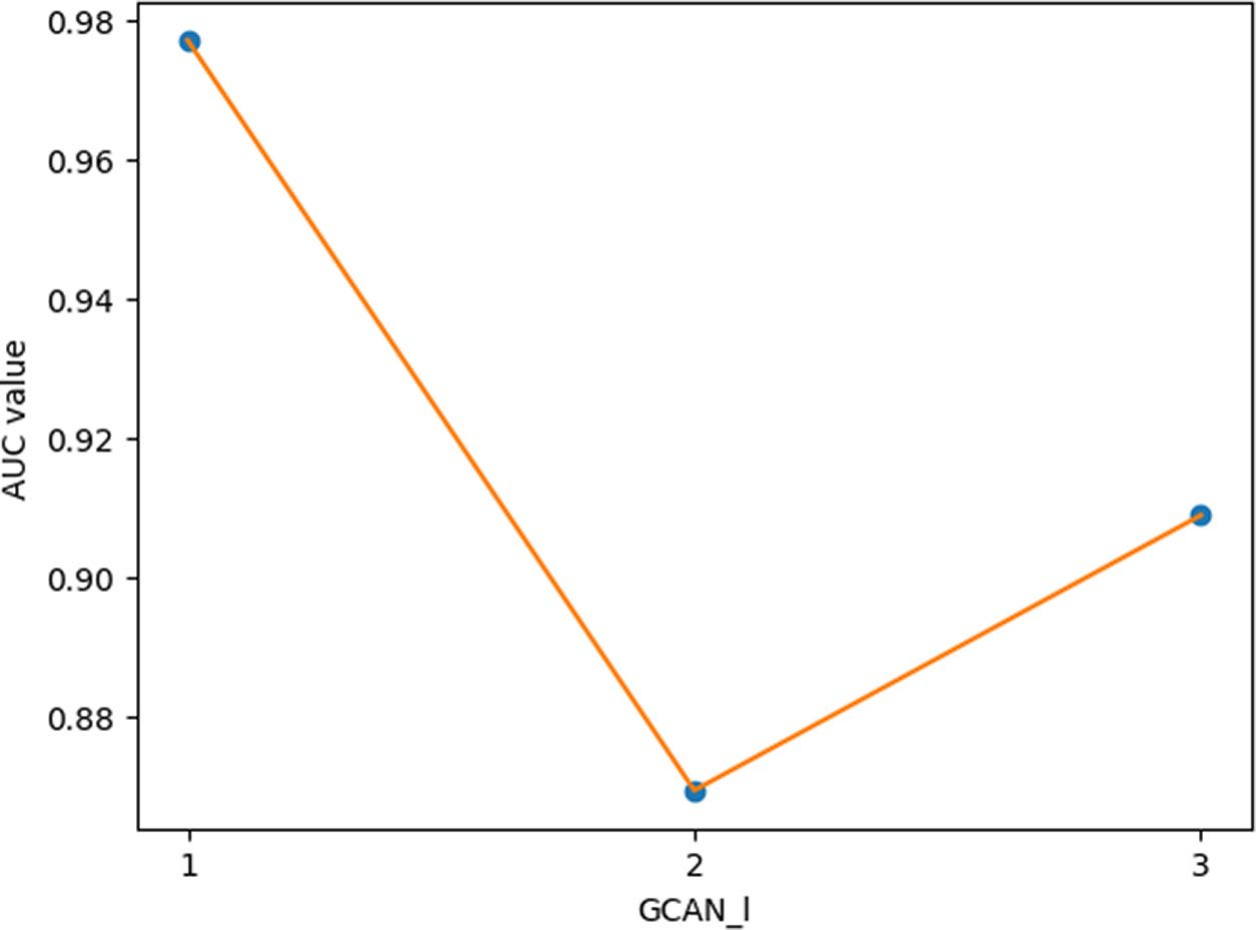
AUCs achieved by GCANCAE with different layers of GCAN.

We further analyzed the effectiveness of components on the prediction performance of GCANCAE and showed the AUCs achieved by GCANCAE without one of these following components such as GCAN, CSAE, or cosine similarity, as shown in Table 4. From observing Table 4, we found that GCANCAE can achieve better prediction performance when adopting both GCAN and CSAE than adopting GCAN or CSAE alone. Moreover, it can improve the prediction performance of GCANCAE by integrating GCAN and CSAE with the cosine similarity as well (Figures 5–10).

**Table 4:** Ablation study.

Models	AUC
CSAE	0.9760
GCAN	0.9765
cosine similarity	0.9429

### 3.3 Case study

In this section, to further evaluate the prediction performance of GCANCAE, we studied the connections between human microorganisms and three types of well-known human respiratory and digestive diseases, such as asthma, obesity, and type 2 diabetes (T2D) based on the HMDAD database and used the publicly available literature to confirm the top 20 predicted microorganisms.

Among these three categories of common diseases, asthma is a heterogeneous disease, accompanied by recurrent wheezing, chest tightness, dyspnea, cough, and other symptoms (Al-Moamary et al., 2021) and has been shown to be closely related to microorganisms (Çalışkan et al., 2013). For example, hemophilia in the lungs of asthmatic patients has been demonstrated to be closely related to the increased risk of neonatal oropharyngeal asthma, and staphylococcus has been found in the respiratory tract of asthmatic children (Sullivan et al., 2016). Table 5 showed the top 20 candidate asthma-associated microbes predicted by GCANCAE, from which it is easy to observe that among these top 20 predicted asthma-related microbes, there are 19 microbes confirmed by previous publications.

**Table 5:** 19 out of the top 20 candidate asthma-associated microbes predicted by GCANCAE have been confirmed by previous publications.

Rank	Microbe	Evidence
1	Betaproteobacteria	PMID:34422359
2	Pseudomonas	PMID:19148935
3	*Prevotella copri*	PMID:28542929
4	*Haemophilus parainfluenzae*	PMID:37287344
5	Coprobacillus	PMID:36245770
6	Paenibacillaceae	PMID:30042764
7	Staphylococcus	PMID:31980492
8	*Staphylococcus aureus*	PMID:31980492
9	Holdemania	PMID:34015282
10	Firmicutes bacterium EG14	PMID: 32072252
11	*Veillonella atypica*	PMID:30561093
12	Actinomyces	PMID:37844548
13	Lachnospiraceae bacterium A2	PMID:31958431
14	*Streptococcus anginosus*	PMID:36741900
15	Bacteroidaceae bacterium Smarlab 3,301,643	Unconfirmed
16	*Clostridium cocleatum*	PMID:16819502
17	Enterococcus	PMID:36451571
18	*Lactobacillus crispatus*	PMID:21108691
19	*Fusobacterium nucleatum*	PMID:35241518
20	*Bacteroides eggerthii*	PMID:37714436

Then, according to statistics, there are currently more than 1.9 billion people obese or overweight in the world. The total prevalence of childhood obesity is 5.0%, and the adult prevalence rate is as high as 12.0% (GBD 2015 Obesity Collaborators, 2017; Saltiel and Olefsky, 2017). Obesity is more likely to cause health complications such as insulin resistance, type 2 diabetes, cardiovascular disease, liver disease, cancer, and neurodegeneration (Saltiel and Olefsky, 2017). Table 6 showed the top 20 candidate obesity-related microbes predicted by GCANCAE, from which, it is easy to observe that among these top 20 predicted obesity-related microbes, there are 19 microbes confirmed by previous publications.

**Table 6:** 19 out of the top 20 candidate obesity-related microbes predicted by GCANCAE have been confirmed by published literature studies.

Rank	Microbe	Evidence
1	Betaproteobacteria	PMID: 30810328
2	Firmicutes bacterium EG14	PMID: 21153634
3	Alistipes	PMID: 30242233
4	Corynebacterium	PMID: 31360527
5	Erysipelotrichales	PMID: 37340959
6	Mobiluncus	PMID: 28177125
7	Promicromonosporaceae	DOI:10.3390/nu10091307
8	Pseudomonas	PMID: 38260892
9	*Staphylococcus epidermidis*	PMID: 33402904
10	*Prevotella copri*	PMID: 36807933
11	*Haemophilus parainfluenzae*	PMID: 38260892
12	*Veillonella atypica*	PMID: 33208788
13	Actinomyces	PMID: 35880087
14	*Clostridium cocleatum*	PMID: 25038099
15	Lachnospiraceae bacterium A2	PMID: 32256098
16	Enterococcus	PMID: 35282803
17	Bacteroidaceae bacterium Smarlab 3,301,643	Unconfirmed
18	*Streptococcus anginosus*	PMID: 32256098
19	Holdemania	PMID: 35382951
20	*Bacteroides eggerthii*	PMID: 34836169

Finally, type 2 diabetes mellitus (T2D), as a complicated chronic condition characterized by hyperglycemia, relative insulin insufficiency, and insulin resistance, has been demonstrated that over 90% of persons with diabetes will have T2D (Sullivan et al., 2016). Common signs and symptoms of T2D include binge eating, excessive drinking, frequent urination, and unexplained weight loss. Although the exact cause of T2D is currently unknown, a combination of lifestyle factors and obesity is likely to be the culprit (Tuomilehto et al., 2001). Table 7 showed the top 20 candidate T2D-related microbes predicted by GCANCAE, from which, it is easy to observe that among these top 20 predicted T2D-related microbes, there are 18 microbes verified by published literature studies.

**Table 7:** 18 out of the top 20 candidate T2D-related microbes predicted by GCANCAE have been confirmed by published literature studies.

Rank	Microbe	Evidence
1	Betaproteobacteria	PMID:29744928
2	Pseudomonas	PMID:26900286
3	Firmicutes bacterium EG14	PMID:20015409
4	*Lactobacillus crispatus*	PMID:32687341
5	*Clostridium coccoides*	PMID: 25784074
6	Bacillus	PMID:20140275
7	*Clostridium cocleatum*	PMID:20857523
8	Firmicutes	PMID: 26595305
9	Clostridia bacterium TSW07CA7	Unconfirmed
10	Bifidobacterium	PMID:32326347
11	*Prevotella copri*	PMID: 36644130
12	*Haemophilus parainfluenzae*	PMID:21741921
13	*Veillonella atypica*	PMID:25926895
14	Actinomyces	PMID:27895859
15	Lachnospiraceae bacterium A2	PMID:31005411
16	Bacteroidaceae bacterium Smarlab 3,301,643	Unconfirmed
17	Enterococcus	PMID:32754068
18	*Streptococcus anginosus*	PMID:33925672
19	*Bacteroides eggerthii*	PMID:30266575
20	*Alistipes finegoldii*	PMID:27760208

In this section, we selected asthma for comparing GCANCAE with the baseline model. During experiments, among the top 15 microorganisms most associated with asthma predicted by GCANCAE and BPNNHMDA, respectively, GCANCAE and BPNNHMDA achieved the same prediction accuracy of 93.3%. Moreover, among the top 20 microorganisms most associated with asthma predicted by GCANCAE and GATMDA separately, the prediction accuracy was 95% for GCANCAE while 90% for GATMDA. Overall, in all microorganisms predicted by GCANCAE, the prediction score of the potential microorganism mostly correlated with asthma was 1.0 and that of the microorganism least correlated with asthma was 0.71.

## 4 Conclusion

The search for treatments and prevention of diseases is crucial when virus-based pandemics are putting human health in risk on a global scale. There is mounting proof that microbes significantly affect human health. Therefore, it is evident that the identification of potential microbe–disease associations from the viewpoint of human microbes and drugs can offer crucial information for comprehending underlying disease mechanisms, which may aid in the study of disease pathogenesis, make early diagnosis easier, and increase the effectiveness of taking drugs.

In this article, we present the GCANCAE model using two models GCAN and CSAE, respectively, to extract the global topology of microbes and diseases and the attribute representations of multiple channels, to predict potential associations between microbes and diseases. Compared with the traditional state-of-the-art methods, the main advance of GCANCAE is to improve the transfer matrix of GCN to pay more attention to the characteristics of the more important nodes. Moreover, the use of multi-channel convolution autoencoder can provide richer feature information, which can help the network to capture more complex data features. Each channel can learn different feature representations, increasing the expression ability of the model. Two different models are used to extract topology and attribute features, which solves the problem that the general model has poor prediction effect on big data and can make better predictions. The results from both comparative experiments and case studies show that GCANCAE outperformed existing representative competing methods and might be a potential efficient tool for future disease prevention. However, while GCANCAE has some advantages over other methods, it has some limitations as well. For example, the convolution channel is time-consuming, and less evidence is used to predict the association between a specific microorganism and a specific disease. To solve the above problems, we will further study and improve the algorithm to reorganize the prediction task based on more public datasets.

## Data availability statement

The original contributions presented in the study are included in the article/supplementary material, further inquiries can be directed to the corresponding authors.

## Author contributions

CZ: Conceptualization, Data curation, Methodology, Software, Writing – original draft. ZZ: Conceptualization, Methodology, Project administration, Resources, Supervision, Writing – review & editing. FZ: Data curation, Resources, Software, Writing – review & editing. BZ: Investigation, Methodology, Visualization, Writing – review & editing. XL: Formal analysis, Software, Validation, Visualization, Writing – review & editing. LW: Funding acquisition, Project administration, Supervision, Writing – review & editing.

## Footnotes

^1^ http://www.cuilab.cn/hmdad

^2^ https://disbiome.ugent.be/
